# AF driver detection in pulmonary vein area by electropcardiographic imaging: Relation with a favorable outcome of pulmonary vein isolation

**DOI:** 10.3389/fphys.2023.1057700

**Published:** 2023-01-30

**Authors:** Carlos Fambuena-Santos, Ismael Hernández-Romero, Rubén Molero, Felipe Atienza, Andreu M. Climent, M S. Guillem

**Affiliations:** ^1^ COR Laboratory, ITACA Institute, Universitat Politècnica de València, Valencia, Spain; ^2^ Department of Cardiology, Hospital General Universitario Gregorio Marañón, Instituto de Investigación Sanitaria Gregorio Marañón (IISGM), Madrid, Spain; ^3^ Centro de Investigación Biomédica en Red de Enfermedades Cardiovasculares (CIBERCV), Madrid, Spain

**Keywords:** atrial fibillation, ECGI, pulmonary vein isolation, clinical outcome, rotors

## Abstract

Pulmonary vein isolation (PVI) is the most successful treatment for atrial fibrillation (AF) nowadays. However, not all AF patients benefit from PVI. In this study, we evaluate the use of ECGI to identify reentries and relate rotor density in the pulmonary vein (PV) area as an indicator of PVI outcome. Rotor maps were computed in a set of 29 AF patients using a new rotor detection algorithm. The relationship between the distribution of reentrant activity and the clinical outcome after PVI was studied. The number of rotors and proportion of PSs in different atrial regions were computed and compared retrospectively in two groups of patients: patients that remained in sinus rhythm 6 months after PVI and patients with arrhythmia recurrence. The total number of rotors obtained was higher in patients returning to arrhythmia after the ablation (4.31 ± 2.77 vs. 3.58 ± 2.67%, *p* = 0.018). However, a significantly higher concentration of PSs in the pulmonary veins was found in patients that remained in sinus rhythm (10.20 ± 12.40% vs. 5.19 ± 9.13%, *p* = 0.011) 6 months after PVI. The results obtained show a direct relationship between the expected AF mechanism and the electrophysiological parameters provided by ECGI, suggesting that this technology offers relevant information to predict the clinical outcome after PVI in AF patients.

## 1 Introduction

Pulmonary vein isolation (PVI) is the most successful treatment for atrial fibrillation (AF) nowadays. Disoite this, 62 months after the intervention only 59% of patients remain free from AF (Kis et al., 2017). The main reason explaining this poor management of AF is the lack of understanding behind the mechanisms that originate and maintain this arrhythmia.

In the last years, many theories supporting localized drivers as the cause of atrial fibrillation (AF) have gained relevance (Guillem et al., 2016; Narayan et al., 2012). Rotors, or functional reentries, are a well-described type of local driver consisting in rotational activation patterns that perpetuate an asynchronous electrical propagation in the heart. Rotors have been theorized and implemented in many *in silico* models (Rodrigo et al., 2017a), as well as observed *in vitro*. However, their identification in the clinics remains elusive. Several factors may hamper rotor identification in the EP lab, like their movement in the atria or the presence of transmural reentrant circuits. However, the main limitation to be faced nowadays is technological. Current sequential mapping systems do not allow for global characterization of the atria, making the identification and tracking of rotors very challenging.

Electrocardiographic Imaging (ECGI) is a non-invasive mapping technology that allows panoramic visualization of the atria, posing it as an interesting tool to understand the presence of rotors in the pulmonary veins (PPVV). This understanding may help to stratify non-invasively patients that may benefit from PVI from those who will return to arrhythmia after the intervention, saving resources and minimizing unnecessary risks for some patients. The most extended methodology used nowadays to localize rotors using ECGI is based on the detection of phase singularities (PSs) in phase maps (Li et al., 2020; Rodrigo et al., 2017b; Molero et al., 2021). Many strategies have been developed so far to identify PSs automatically (Li et al., 2020). However, the lack of standardized postprocessing techniques to identify PSs in phase maps obtained from ECGI and technological limitations associated to ECGI validation have raised skepticism on the use of phase maps and confusion about the electrophysiological interpretation of phase singularities (PS) (Podziemski et al., 2018; Cluitmans et al., 2018).

In this context, the main objective of this article is to develop and validate a methodology to identify reentries in AF patients using ECGI data. Additionally, the capacity of ECGI to find differences between patients in which the pulmonary veins region plays a relevant role in the maintenance of AF, and those in whom other atrial zones could be responsible of AF maintenance was studied.

## 2 Materials and methods

### 2.1 Study population

The data acquisition protocol used in this study was approved by the ethics committee of Hospital Gregorio Marañón, Madrid, Spain (reference 475/14) and all patients provided informed consent of the procedure. A total of 29 AF patients participated in the study with a subpopulation of 7 men and 22 women (mean age of 62.63 ± 14.26). The number of patients presenting paroxysmal and persistent AF numbered 16 and 13, respectively. Antiarrhythmic drugs were taken by a set of 6 patients before PVI procedure (for more details, see Table 1).

**TABLE 1:** Description of the population used in the study.

		All patients (*n* = 29)	Non-recurrence (*n* = 15)	Recurrence (*n* = 14)
Male (%)		7 (24.14%)	6 (40.00%)	1 (7.14%)
Age (Years)		62.63 ± 14.26	58.13 ± 15.06	64.57 ± 13.18
Paroxysmal AF (%)		16	11 (73.33%)	5 (35.71%)
Valvuloplast**y** (%)		14	8 (53.33%)	6 (42.86%)
Medical Therapy	Beta-blockers	1	1	0
	Flecainide	1	1	0
	Amiodarone	6	3	3
Medical Therapy After Ablation	Beta-blockers	15	5	10
	Flecainide	2	2	0
	Amiodarone	12	5	7
Patients with Previous Ablation		27 (93.10%)	14 (93.33%)	13 (92.86%)
Ablations Per Patient		1.14 ± 0.51	1.13 ± 0.52	1.14 ± 0.53

Patients were split into 2 groups attending to their clinical outcome 6 months after PVI. Those who remained in sinus rhythm were labelled as “Non-recurrence” patients (*n* = 15) and those that presented any kind of arrhythmia (i.e., AF or Atrial flutter) were classified as “recurrence” (*n* = 14). Clinical validation of the cardiac rhythms 6 months after the procedure was performed with a standard 12 lead ECG and quality-of-life questionnaires (Molero et al., 2021).

### 2.2 Ablation and electroanatomical mapping

Circumferential PVI was performed in all 29 patients point by point using irrigated catheters (Cool-Flex/TactiCath/Sapphire-Blue, St. Jude Medical). Energies ranging 25–35 W were delivered *via* conventional ablation in both pairs of pulmonary veins. Additionally, FIRMap 64-poles mapping catheters were consecutively placed in the right and left atrium for 3-dimensional electroanatomic imaging (NavX, St. Jude Medical). Cather placement was achieved *via* vein access through the femoral vein reaching the right atrium and later to the left atrium through transeptal punction. In patients arriving in sinus rhythm electrical burst pacing was used to induce AF (Rodrigo et al., 2020).

### 2.3 Data acquisition and preprocessing

Body Surface Potential Maps (BSPM) were recorded in all the patients during AF right before circumferential PVI. A set of 57 electrodes were employed to record the signals. The acquisition and preprocessing protocols employed in this study were already presented previously (Molero et al., 2021; Rodrigo et al., 2020). As an overview, BSPM signals were recorded with a 1 kHz sampling frequency and filtered using a 0.05–500 Hz bandpass filter. The geometry of the torso and electrode positions were determined using photogrammetry and the anatomy of the atria was segmented from MRI/CT images. A total of 57 segments with similar durations (4.06 ± 0.311 s) were included in the analysis. Additionally, other 57 segments were recorded during adenosine injection in order to compare the clinical results obtained from adenosine-free signals (see Supplementary Material S1). Regarding signal processing, the baseline was removed using a low-frequency polynomial fitting. The signal was then filtered using a 10th Butterworth band-pass filter with cut-off frequencies of 2 and 45 Hz. In order to remove the ventricular activity, QRS cancellation was performed following a temporal PCA separation approach (Castells et al., 2005). The inverse problem was then computed using the boundary element method formulation, and zero-order Tikhonov regularization with L-curve optimization methods for matrix inversion. Once the 3D voltage maps were obtained, these were transformed into 2D squared images using a Teichmüller external mapping approach, guaranteeing a uniform conformal distortion of the 3D point cloud surface (Meng et al., 2016) (See Supplementary Material S1 for more information). Finally, phase maps were computed from 2D voltage maps by means of the Hilbert transform.

### 2.4 Rotors and phase singularities detection

The details on the proposed rotor detection algorithm are explained in Supplementary Material S1 but the general workflow of the algorithm is described in this section. The first stage on the detection algorithm consists on the identification of the center of rotation of rotors, namely phase singularities (PSs), on a given time instant. In this study, PS are defined as locations in the atria surrounded by the whole activation cycle, or equivalently, the full range of phases [-π,π] obtained by applying the Hilbert transform on inverse computed electrograms. PSs are identified by the topological charge (Bray and Wikswo, 2002). Then, PSs non-complying with a gradual spatial progression of phases between -π and π in their surroundings (Rodrigo et al., 2017b) were discarded. After having identified PSs we then connect them spatiotemporally into rotors and through filaments in order to both discard transient PSs that appear for a short period of time most likely corresponding to changes in direction of the propagation front- and connect PSs whose tracking is transiently lost. Rotors filaments were then dilated using a prism-shaped kernel in the direction of the trajectory, and skeletonized in order to cluster and identify the trajectories. Finally, the number of turns is quantified in each rotor and PSs belonging rotors that spin for less than a given number of turns are discarded for further analysis.

### 2.5 Rotor metrics and clinical outcome study

Two different parameters were calculated to study the relationship between the detected reentrant activity, and the clinical outcome of the patients after PVI. The first parameter was the number of rotors or clusters in the whole atria per unit time. The second set of parameters was the percentage of PSs at 9 different atrial regions. This percentage was calculated as the number of PSs detected at a particular atrial region over the total number of PS detected. After clustering PSs into rotors, the mentioned metrics were obtained in two different conditions: Considering all the detected rotors and PS, or keeping only those rotors (and correspondent PSs) that spin for at least 1 turn. These two implementations were applied to recordings obtained with and without adenosine injection. Finally, Mann-Whitney U test was used in order to compare the number of rotors detected in ‘Non-recurrence’ and “Recurrence” groups. The same test was used to study statistical differences in the percentage of PS obtained in the 9 different atrial regions. An independent test was used to evaluate differences between ‘Recurrence’ and “Non-recurrence” groups in each region using only values obtained from different recordings, assuring in this way, independency between samples.

### 2.6 Validation dataset

The proposed methodology for rotor detection in ECGI data was evaluated both, using recordings from patients and simulated data. Specifically, two mathematical models with well-defined location of reentries were employed. To identify the reentries maintaining the arrhythmia, we performed a virtual ablation in the middle of the fibrillatory episode. Then we solved the inverse problem and applied the rotor detections algorithm presented in this study to compare the pre- and post-ablation scenarios (see Supplementary Material S1 for further details on the simulations).

Regarding the validation in patients, a dataset of 9 ECGI segments where reentrant patterns could be visually identified were selected from the main database of patients. The algorithm was applied in these recordings in order to detect rotors, which were blindly labelled in the validation dataset according to two conditions: rotors spin for at least 1 turn and there was linear progression of the phase around the detected singularities. These conditions were visually checked by the operator labelling the data. The detected and manually labelled set of detections were then compared.

### 2.7 Evaluation metrics

The performance of the algorithm was evaluated using the precision (P) and recall (R), defined in Eqs 1, 2:

$$P=\frac{TP}{TP+FP}$$ (1)

$$R=\frac{TP}{TP+FN}$$ (2)

Where TP are the true positive detections, FP the false positive and FN the false negative. Precision (P) is a measure of how much the proposed algorithm overestimates the presence of PSs. On the other hand, recall (R) measures how often PSs are undetected. All the PSs detected within a tolerance distance of 5 mm from a manually labelled PS were considered true positives. In contrast, false positives were annotated when more than one PS was detected in the 5 mm surrounding area of a labelled PS. False negatives were identified as the absence of any detected PS withing a 5 mm distance from a labelled PS. In order to include the precision and recall information in only one parameter, the Fβ score was also computed [5] (see Eq. 3). This parameter allows to weight the relevance of the precision and recall in the final score through the constant β. In this study the same value presented in the literature of β = 2 was used for better comparison.

$$F_{\beta }=\left(1+\beta^{2}\right)\cdot \frac{P\cdot R}{\beta^{2}\cdot P+R}$$ (3)

Finally, the regional accuracy of the algorithm was also evaluated. For that, the atrial geometries of all the patients were divided into 9 different regions depicted in Figure 3. The number of PS manually labelled in the validation data set was then compared to the number of PS detected by the algorithm in every region. The mean absolute error between these two quantities and the relative error were calculated. This last was defined as the ratio between the mean absolute error and the mean number of PSs labelled in each region.

## 3 Results

### 3.1 Validation of the rotor detection algorithm in simulations

**FIGURE 1:**
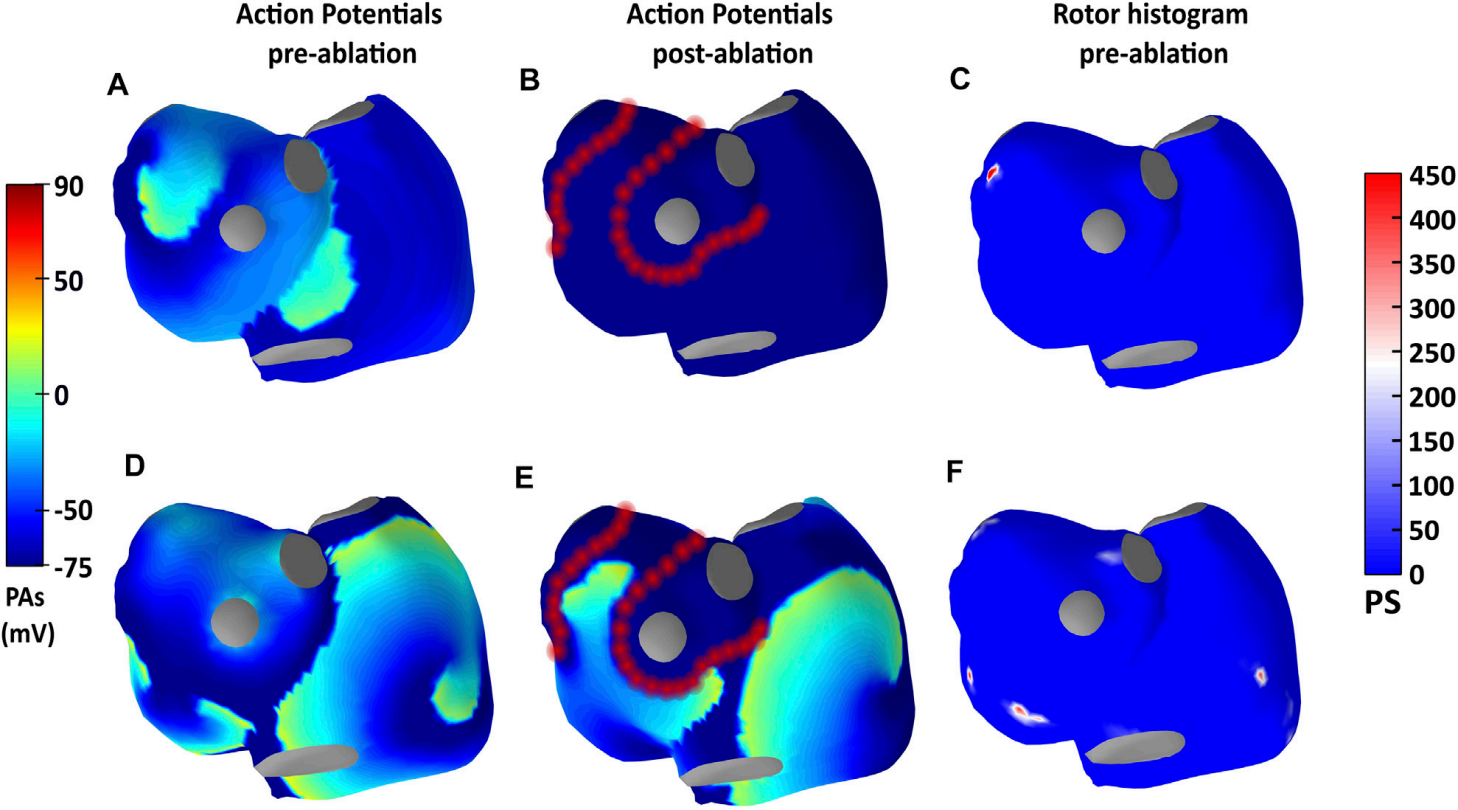
Information about simulations 1 and 2 is presented in the first and second panel rows. Panels **(A, D)** show the simulated action potentials in an instant previous to the ablation, while panels **(B, E)** show the action potentials after ablation. In **(C, F)**, ECGI-derived rotor histograms of the pre-ablation periods are shown.

The simulated AF episodes are illustrated in Figure 1. In the first AF episode (Figures 1A–C), a reentry driving the arrhythmia was localized near the left pair of PPVVs. In Figures 1A, B the transmembrane potentials of two different pre- and post-ablation instants in this episode are presented. The reentry was identified in ECGI signals by using our rotor detection algorithm after forward and inverse propagations. The rotor histograms summarizing the whole reentrant activity in the pre-ablation period is shown in Figure 1B. We can observe how most of the reentrant activity is located near the left pair of pulmonary veins. A virtual ablation in the PPVV area was able to stop the arrhythmia (Figure 1C). In contrast, in the second episode (Figures D–F) reentrant activity is spread throughout different atrial locations far from the PPVV. When applying PVI, the electrical activity is prevented from entering in the PPVV area and the dynamics of the arrhythmia are modified (see Figure 1F). However, PVI is not able to eliminate rotors present in the right atrial posterior wall, leaving some pro-arrhythmic mechanisms intact outside the PPVV and, thus the arrhythmia is not terminated.

### 3.2 Validation of the rotor detection algorithm in patients

**FIGURE 2:**
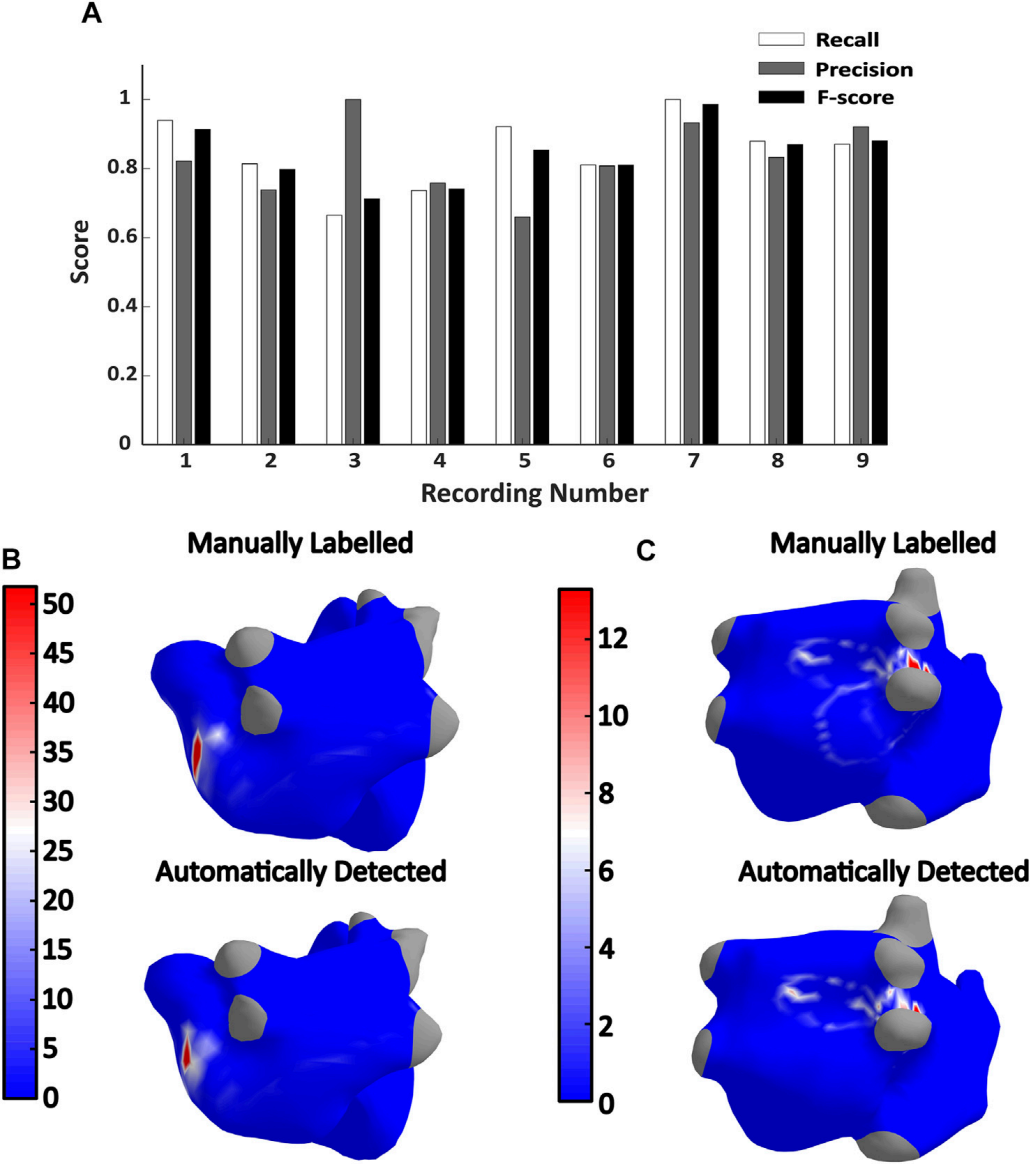
**(A)** Recall, precision and F-score values obtained in the validation dataset. **(B,C)**, Manually labelled and automatically detected rotor histograms obtained from recordings 9 (top) and 3 (bottom) respectively. The recall, precision and F-score values calculated in recording 9 were 0.87, 0.92, and 0.87 while the recall, precision and F-score values of recording 3 are 0.66, 1, and 0.7 respectively.

In Figure 2A the global recall, precision and F-score values obtained for all the recordings in the validation dataset are represented. The mean F-score, precision and recall values of all these recordings were 0.84, 0.84 and 0.85 respectively. The lowest recall was found in recording 3 with a value of 0.66, and the lowest precision was found in the recording number 5 with also a value of 0.66. In Figures 2B, C a comparison of the rotor histograms obtained in two different recordings are presented: recording 9 with a high recall value (0.87), and recording 3 with a low value (0.66). In this example, it is shown how, in spite of missing some of the reentry areas (mostly in the right pair of PPVV), a map with very similar locations of the main reentrant activity is obtained.

**FIGURE 3:**
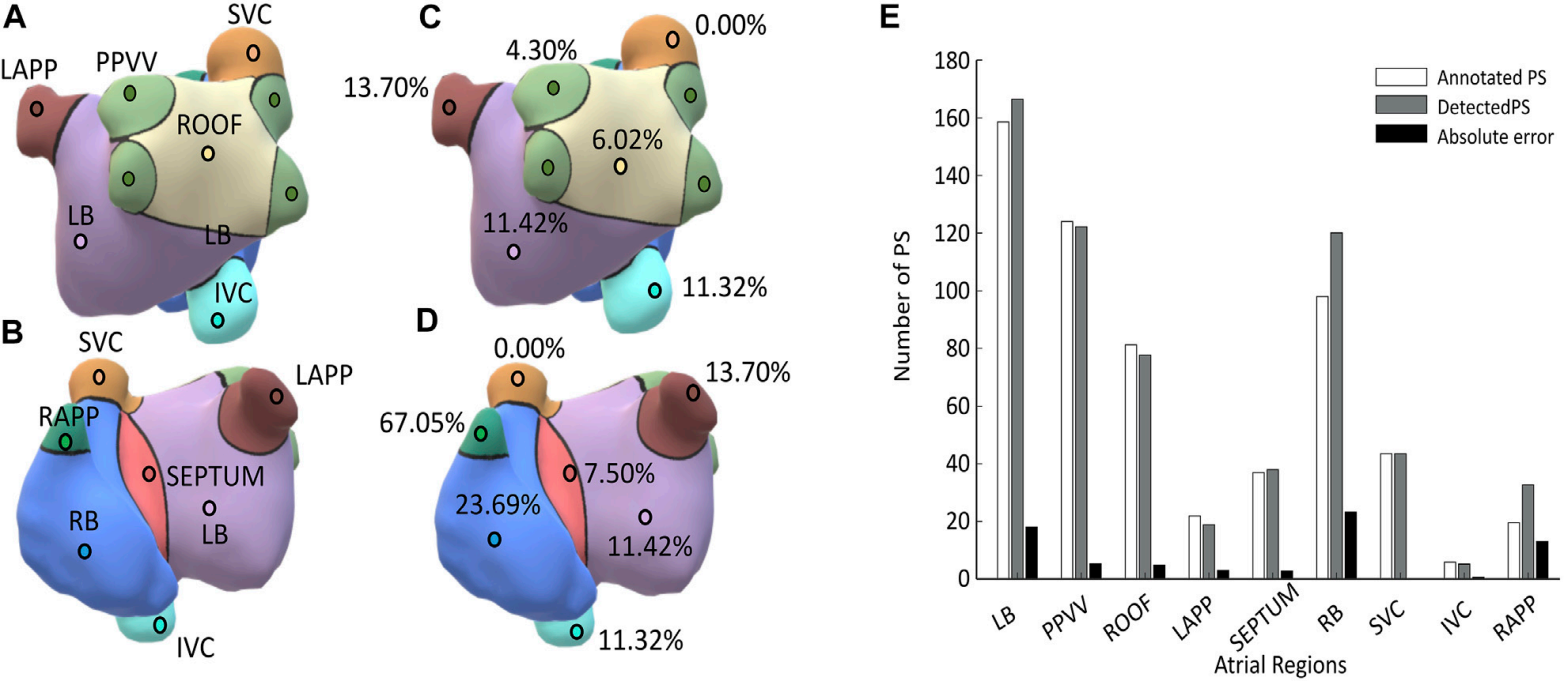
**(A)** and **(B)** Illustration of the regional anatomical division of the atria; **(C)** and **(D)** relative errors obtained in each of the atrial regions; **(E)** Mean number of annotated and detected PSs in different atrial regions across the validation dataset. The mean absolute error between these two magnitudes is also presented in black.

The accuracy of the algorithm was also evaluated regionally. The proposed division for the atria is shown in Figures 3A, B. Regarding the local accuracy in each of these regions, the mean number of detected and annotated PSs along with the mean absolute error between these two magnitudes are presented in Figure 3E. In general, it can be observed a good correspondence between the annotated and detected PSs with a mean absolute error of 7.9 PS across all the regions. The areas with higher absolute errors were the RB (23,22 PSs) followed by the LB (18,11) which are also the largest regions in the proposed atrial segmentation. Regarding the relative error, it ranged between 0% and 67% with a mean value of 16.11%. The SVC is the atrial structure with lower relative error and RAPP the one with the largest. The distribution of the relative errors in the rest of atrial regions is presented in Figures 3C, D.

### 3.3 Relation between rotors and PVI outcome

**FIGURE 4:**
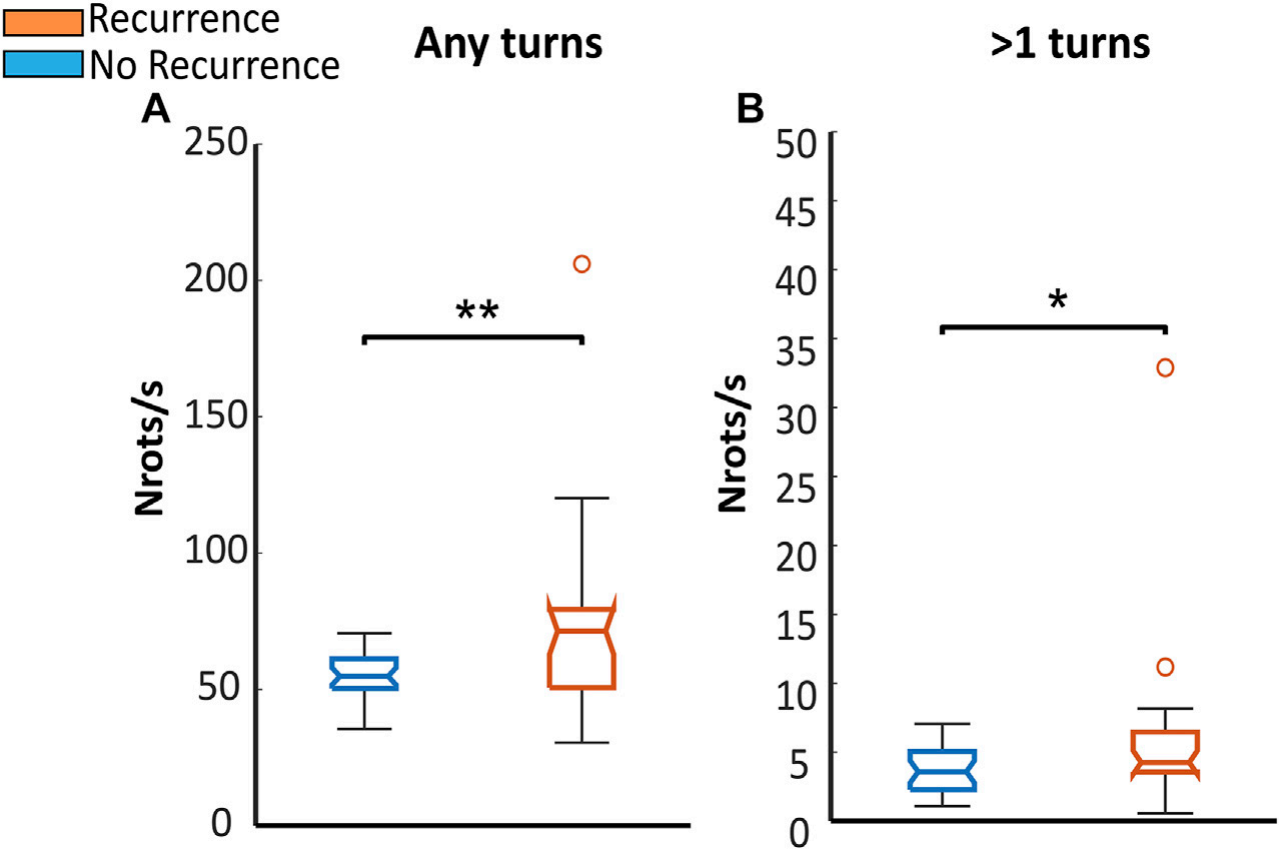
Number of rotors obtained in all the patients: In blue are represented patients staying in sinus rhythm (No recurrence) and in orange patients returning to arrhythmia 6 months after PVI (Recurrence). The number of rotors was calculated in the whole atria in two different scenarios regarding the number of turns used to filter the PS: **(A)** Number of rotors obtained without any turn filtering; **(B)** Number of rotors spinning for at least one turn.

**FIGURE 5:**
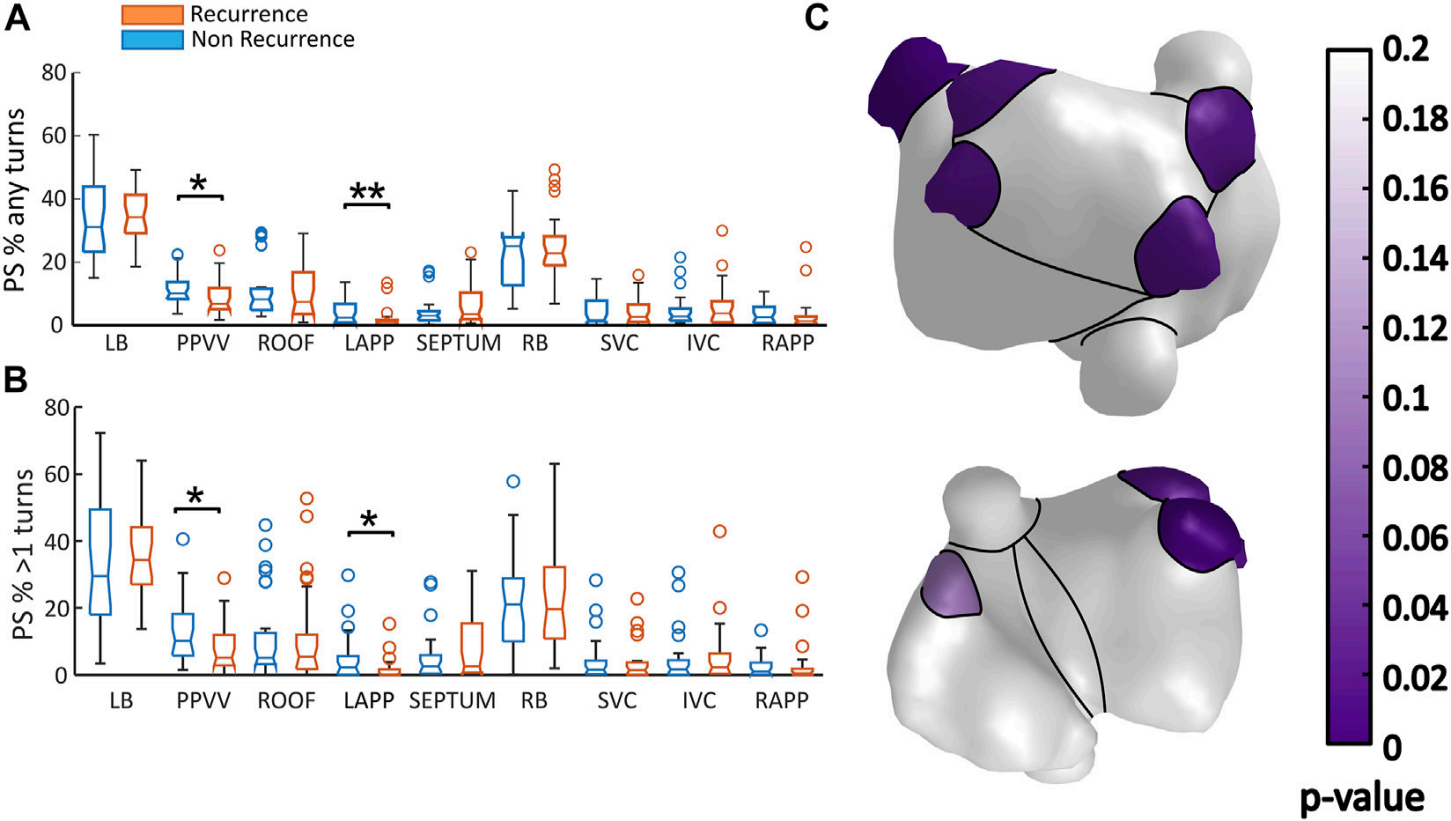
**(A,B)** Proportion of PSs per second detected in different regions in each studied group: in blue is presented the data from patients that remained in sinus rhythm 6 months after ablation (Non-recurrence), and in orange patients that returned to arrhythmia (Recurrence). In panel **(A)**, are presented the results obtained when all the reentries are considered in the analysis. In panel **(B)**, the same results are shown, when only the reentries lasting for more than 1 turn. The distribution of the statistical *p*/values obtained for each regions are presented din panel **(C)**.

In Figure 4 the number of rotors per second obtained in the whole atrium is presented for both studied groups: patients that remained in sinus rhythm and patients that returned to arrythmia 6 months after PVI (AF-recurrence). The number of rotors was quantified in two different ways: including all the rotors detected (any turns, Figure 4A) and discarding those rotors spinning for less than 1 turn (Figure 4B). Patients with an unsuccessful PVI had more rotors in the whole atria than patients remaining in sinus rhythm 6 months after the ablation. This difference was more noticeable when all the rotors detected were considered in the analysis, median values 54.83 ± 10.43 vs. 70.92 ± 27.77 (*p* < 0.01, Figure 4A). However, when keeping only rotors that spin for at least 1 turn, the difference is still preserved although the significance lowers down (3.59 ± 2.67 vs. 4.31 ± 2.78, *p* < 0.05, Figure 5B).

In both groups, higher concentrations of PS are found in the regions located in the left atria. This result is in line with the general belief that most of the initiation mechanisms of AF are present in the left atrium. In a regional level, a larger proportion of PS in the PPVV was always found in the non-recurrence group. This last finding is expected given the assumption that the mechanisms driving AF are more frequently located in the PPVV in patients with a successful PVI. This difference is presented in Figure 5B for stable reentries, that is, reentries spinning for at least 1 turn (10.20 ± 12.40 vs. 5.19 ± 9.13, *p* > 0.05), but also in Figure 5A where all the PS detected are included in the analysis. The statistical differences obtained in the different atrial regions are presented in Figure 5C, where it is shown that the PPVV and LAPP areas are the only areas presenting *p*-values lower than 0.05.

When considering all the PSs and not only those completing a full rotation, sudden wavefront direction changes due to conduction block areas, wavefront collisions or partially detected transmural reentries may be detected as PS. These factors may be indicators of the complexity of the arrhythmia and the underlying atrial substrate. However, also far field artefacts may be detected as PSs and included in the analysis. Under these conditions, it is interesting to remark that the same regions presenting statistically significant differences in the proportion of PS remained, including the PPVV (10.13 ± 5.47% vs. 6.81 ± 6.71%, *p* < 0.05) and LAPP, being the proportion of PS larger again in the non-recurrence group of patients.

## 4 Discussion

In this study, a rotor detection algorithm was presented and validated. Additionally, a higher prevalence of PS prevalence in the PPVV area in patients with a favorable outcome after PVI was found with this algorithm. This finding suggests that the identified reentrant activity is linked with the mechanism sustaining AF.

### 4.1 Algorithm evaluation

In terms of global performance, the use of simulations showed a precise localization of the main reentrant activity during AF as well as a mechanistic explanation on why PVI works in the first simulation, with a localized reentry in the PPVV area, and not in the second, with multiple spread drivers. Regarding labelled data coming from AF patients, it was found that the F-score value obtained (0.84) outperforms other algorithms presented in the literature. A closely related study in this regard is (Li et al., 2020), where the same definitions of precision, recall and F-score values were used. In this study, 4 different rotor detection algorithms were tested in 2D geometries obtaining F-scores values ranging between 0.527 and 0.831. Similarly, these algorithms were validated in non-invasive ECGI phase maps obtained from AF patients, where the presence of rotors was manually labelled by an operator.

The performance of our algorithm was also evaluated locally in 9 different regions. The mean discrepancy present in all areas between the detected and annotated PSs was 16.11%. An abnormally high relative error in the RAPP region (67%) was observed. The origin of this high error must be related to the low number of PSs detected in the RAPP. This factor increases the effect of miss detections in the relative error value.

### 4.2 ECGI in the detection of AF sources

The existence of well localized mechanism driving AF has been reported by means of invasive and non-invasive mapping techniques (Narayan et al., 2012; Haïssaguerre et al., 2006; Gao et al., 2019). This finding has justified the use of personalized driver-specific ablation strategies to terminate AF. In this line, Narayan et al. (2012) showed that focal impulse and rotor modulation ablation (FIRM), in combination with PVI, had a higher successful rate in long-term AF termination than standard PVI alone. Haissaguerre et al. (2014), also tried a similar ablation strategy, but this time obtaining the cumulative maps of focal and reentrant activity from ECGI mapping. They guided ablation in 103 patients with persistent AF demonstrating that the mean ablation time can be reduced guiding the procedure with non-invasive ECGI mapping. Furthermore, a higher degree of fractionation in reentry areas identified with ECGI was found, providing clinical evidence of the detections.

In spite of the clinical evidence showing the potential advantages of a personalized ablation strategies, the positive results obtained in the mentioned studies have not been reproducible. A possible reason for this may be the lack of robustness of the overall approach and a dependence on the operator expertise to determine what is a reentry. In our pipeline, we include a full methodology that allows to remove the human factor by automatically computing the number of turns of the rotors and filtering the PSs with no linear progression of phases in their surroundings.

### 4.3 PS detection and rotor tracking with ECGI

We have already developed automatic PS detection methods for ECGI in the past. Specifically, Rodrigo et al. (2017b), Rodrigo et al. (2017a) proposed a methodology to detect reentries directly on 3D atrial meshes. There, PS were detected at nodes in which the full range of phases [-π,π] was observed in their surroundings. Additionally, the spatial progression of the phases was checked to be ‘linear’ around each PS. The resultant PS were then tracked to obtain filaments or rotors by imposing a radius distance criterium. This methodology was validated in AF simulations.

Other studies have proposed to detect reentries by means of local activation time (LAT) mapping (Vandersickel et al., 2019; Rogers et al., 1999). However, this approach is hardly transferable to ECGI during AF since there is no consistent method for detecting LATs in ECGI signals during AF. Some challenges causing the lack of standard methods are the inherent spatial smoothing present on ECGI electrograms and the presence of remaining far-field artifacts leading to loss of tracking of PSs that needs to be corrected. In this context, phase mapping has been postulated as an alternative way to detect reentries in ECGI (Haissaguerre et al., 2013; Dhillon et al., 2021). Phases provide equivalent information about the electrical propagation encoded in the low-frequency content of the signals with no need for LAT detection. Given the exposed advantage, we decided to stick to a phase-mapping based approach, but updating our methodology according to the results obtained in (Li et al., 2020). In this work, algorithms based on topological charge, a metric closely related to the curl of the electrical propagation, presented the highest performance for PS detection. In addition, around the PSs obtained from topological charge, a checkup of linearity in the surrounding phases was included. This feature overcomes the presence of false positive PS at locations with complex or fractionated signals (Rodrigo et al., 2017b). Additionally, we perform PS detections in a 2D domain in order to speed up the calculations. For that, we use a transformation that assures a homogeneous distribution of nodes in the atria, and provides a direct transformation of the data to a 2D grid, where we can efficiently track rotors. Furthermore, having a homogeneous disposition of nodes is also very convenient in some steps of our methodology like phase linearity checking around PSs, or counting the number of turns per rotor. Other 2D-3D transformations, such as the universal atrial coordinates (UAC) system have been proposed in the past (Roney et al., 2019). However, our methodology is better suited for our purpose than UAC. First, our transformation is fully automatic and does not require from manual landmark placement. Additionally, UAC was conceived for data transferring between different patients and does not result in a uniform grid with preservation of distances in the original mesh.

The methodology for rotor tracking was also updated in this study. It consists in combining neighbor voxels containing a PS into filaments. The methodology is completely based in image processing operations such as voxel dilation and skeletonization which present two main advantages with respect to the previous approach. The first one comes from the efficiency of convolutional operations such as dilation or skeletonization performed on image stacks. The second one is about specificity. Our tracking algorithm is more specific when combining PS into rotors than imposing a radius around each PS in space. This is because a prism-shaped dilation kernel is applied following the direction of the trajectory of the rotor at each voxel, preventing the algorithm from combing independent filaments together that eventually get too close in space. A similar methodology was applied in (Clayton and Holden, 2004), where filaments in simulated ventricular fibrillation episodes were created by linking together voxels in a 3D space. In contrast with our algorithm, the method used in Clayton et al. uses a grassfire algorithm instead of dilation and skeletonization operations to obtain filaments. This approach has the advantage that no kernel needs to be specified like in our approach, however the computational time needed to run it is higher. Finally, another important difference between our method and the one employed by Clayton et al. is that we count the number of turns of each rotor by taking into account the surround phases of the filament. This last step allows us to filter out rotors with a low number of turns.

### 4.4 Non-invasive atrial substrate evaluation in AF patients

The results obtained in this study suggest that ECGI provides relevant information about the functional state of the atria in AF patients. In a prior study we already related the stability of rotor-derived metrics with ablation outcome (Molero et al., 2021) while we could not relate the location of these rotors with the success of PVI, in the same line as other studies (Dhillon et al., 2022). In this study we have developed a new methodology for rotor tracking that allows for a more restrictive definition of a rotor, specifically suited for analysis of ECGI data, a context with low spatial resolution and loss of tracking due to remaining far-field components. In addition to the methodological differences for PS detection and rotor tracking explained in the previous section, we also explored regional differences in the presence of reentries. For that purpose, we divided the atria in 9 different areas where the prevalence of reentries was estimated as the percentage of PS. Additionally, we computed a different global parameter, the total number of rotors detected in the atria per unit time.

Regarding the number of rotors per second found in the atria, a larger number was detected in the recurrence group, reflecting a possible higher complexity of the electrical activation patterns in patients with poor PVI outcome. This finding is in concordance with the reproducibility results obtained by Molero et al. A possible explanation supporting this findings is the detection of changes in the direction of wavefronts as PS. Relative to this, Podziemski et al. (2018) stablished a relationship between PSs obtained from phase maps and spots of conduction block. In this way, recurrence patients with a higher prevalence of complex atrial substrate may have more conduction block spots that produce this effect. Another explanation is the detection of hidden transmural rotors where only part of the rotor cycle is visible in the epicardium. In this regard, Clayton and Holden. (2004) observed in *in silico* simulations of ventricular fibrillation (VF) that only 65% of reentries touched the epicardium and most of them for less than one cycle.

**FIGURE 6:**
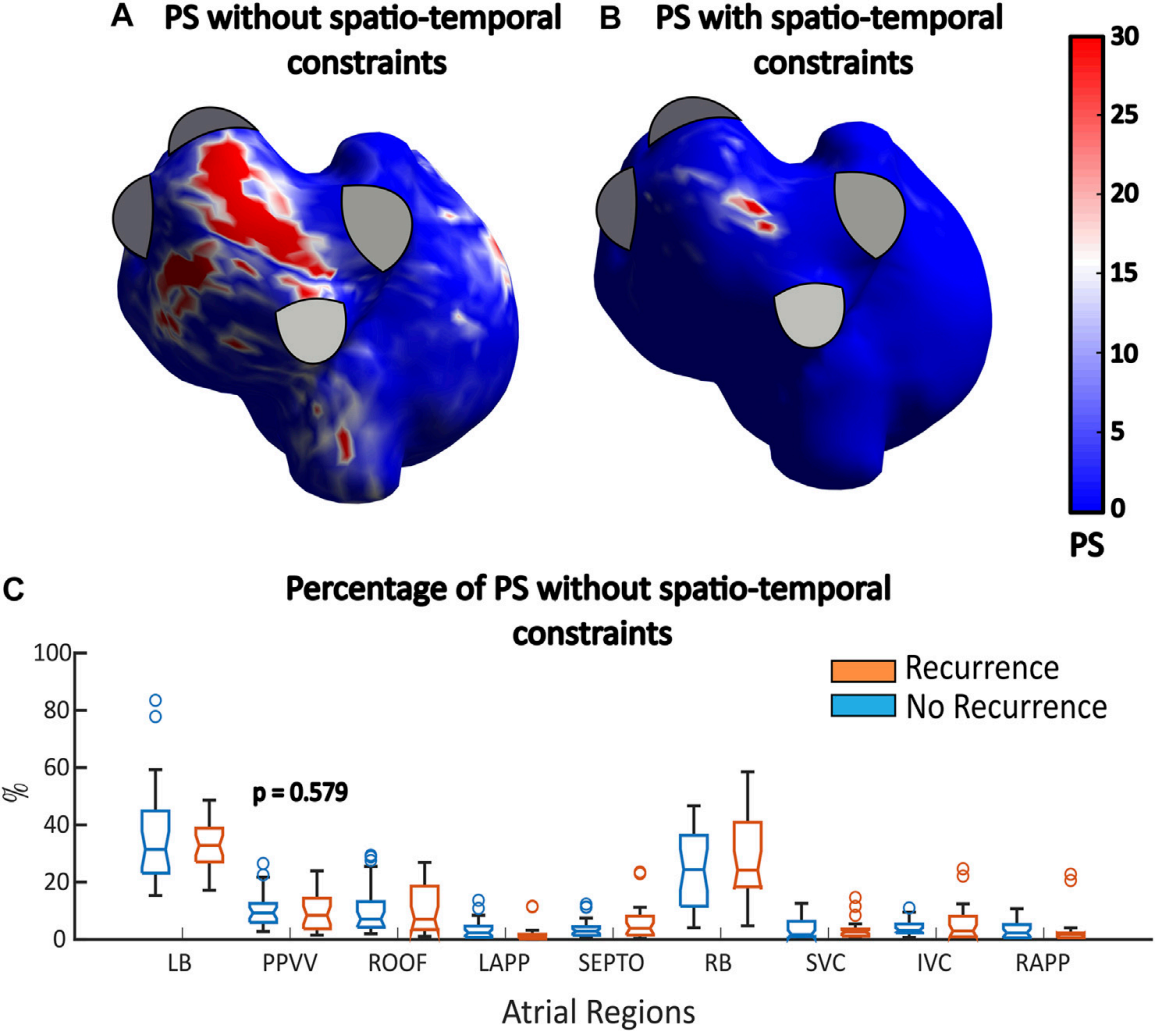
**(A,B)** PS histograms with and without filtering. **(C)** Percentage of PSs found in all the different atrial regions when no filtering strategies are applied. No statistically significant differences are observed.

In addition to factors that are actually related to the dynamics and complexity of the arrhythmia, the presence of far field artefacts and the limited spatial resolution reduce the accuracy of ECGI to provide the exact location of reentries. In this scenario, postprocessing algorithms employed on top to the ECGI signals are of great importance to detect rotors robustly. Indeed, some studies quantifying the amount reentries in the PPVV have found a great overlap between recurrence and non-recurrence AF patients using CardioInsight system (Dhillon et al., 2022; Gao et al., 2019), In our study, we also observed how the number of rotors overlapped between the studied groups. However, with our methodology, in contrast with the previous ones, we obtained statistically significant differences in the number of rotors and the proportion of phase singularities in the PPVV. In Figure 6 it can be observed how when keeping only PS with a linear progression of phases in their surroundings, the areas showing a high prevalence of reentries shrinks down. In this example, red areas of high PS prevalence outside the roof of the atria are removed, keeping only a small core. This filtering has therefore an effect on the percentage of PS calculated in the different areas. When comparing these percentages in the recurrence and non-recurrence group of patients with no filtering strategy, we see how all the statistical differences observed with our post-processing algorithm disappear, with a *p* = 0.579 in the PPVV. This observation highlights the importance of the methodology to observe differences in the reentrant activity when comparing recurrence and non-recurrence AF patients after PVI.

Finally, a higher prevalence of PSs in the PPVV area was quantified in patients with a favorable outcome after PVI. According to our results, the application of a threshold of one turn enhances the differences in the prevalence of PSs in the PPVV area as compared to other regions. In this regard, it was observed how the significant difference found in the LAPP obtained when including all the PS in the analysis (no turn filtering), was reduced when only PS belonging to long-lasting rotors (1 turn or more) are included in the analysis. A possible explanation for this reduction is the role of the LAPP as an anatomical obstacle, leading to direction changes in the propagation of wavefronts that do not cover an entire turn. In this way, the application of a threshold of 1 turn seems to reduce the relevance of this secondary patterns in the analysis, enhancing the importance of the mechanisms present in the PPVV. Additionally, this analysis was also performed during adenosine injection (see Supplementary Material S1). Adenosine is a drug that stops the ventricular activity, providing a more favorable scenario for the inverse problem. Again, similar tendences and significant differences in the proportion of phase singularities were found between the studied groups. The coherence in the results obtained under different filtering and adenosine conditions suggest that the presented approach is robust against far field artefacts coming from the ventricular activity.

### 4.5 Clinical implications

The efficacy of PVI in AF patients decreases as the disease progresses due to the higher complexity and variety of mechanisms involved in the arrhythmia (Haïssaguerre et al., 2006; Haissaguerre et al., 2014). This progression leads to a lower success of PVI in patients with an advanced disease. In this study, we observed that the prevalence of reentries measured as singularity points in non-invasive phase mapping is a predictive factor of the clinical outcome of patients undergoing PVI. Therefore, this finding can help clinicians to stratify patients non-invasively before any electrophysiological study, saving in this way resources and reducing risks for patients who will not benefit from PVI.

### 4.6 Limitations and future work

Some of the characteristics of the dataset employed, such as the large prevalence of previous ablations or valvuloplasties are very particular of our population. Additionally, due to the lack of a standardized gold-standard in rotor detection with ECGI technology, we have opted for manually labelling ECGI data, which may somehow bias our results. In the future, we plan to continue the validation of the algorithm presented in this study, by comparing the locations of reentries to low voltage areas obtained with electroanatomical mapping.

## 5 Conclusion

In this study we have shown that ECGI allows detecting the spatial dominance of the reentrant activity at different atrial regions during AF episodes. A higher concentration of PSs in the PPVV was found in those patients that remained in sinus rhythm 6 months after PVI, showing a direct relationship between the expected AF mechanism and the electrophysiological parameters provided by ECGI.

The results of this study may help in the future to predict which patients will benefit from a PVI procedure. Furthermore, we have shown that the approach employed is robust and valid for non-invasive applications like personal AF studies or patient stratification.

## Data Availability

The original contributions presented in the study are included in the article/Supplementary Material, further inquiries can be directed to the corresponding author.

## References

[B1] Bray M. A. Wikswo J. P. (2002). ‘Use of topological charge to determine filament location and dynamics in a numerical model of scroll wave activity’. IEEE Trans. Biomed. Eng. 49 (10), 1086–1093. 10.1109/TBME.2002.803516 12374332

[B2] Castells F. Mora C Rieta J. J. Moratal-Pérez D. Millet J., (2005). ‘Estimation of atrial fibrillatory wave from single-lead atrial fibrillation electrocardiograms using principal component analysis concepts’. Med. Biol. Eng. Comput. 43 (5), 557–560. 10.1007/BF02351028 16411627

[B3] Clayton R. H. Holden A. V. (2004). ‘Filament behavior in a computational model of ventricular fibrillation in the canine heart’. IEEE Trans. Biomed. Eng. 51 (1), 28–34. 10.1109/TBME.2003.820356 14723491

[B4] Cluitmans M. Brooks D. H. MacLeod R. Dossel O. Guillem M. S. van Dam P. M. (2018). ‘Validation and opportunities of electrocardiographic imaging: From technical achievements to clinical applications’. Front. Physiology 9 (), 1305–1319. 10.3389/fphys.2018.01305 PMC6158556 30294281

[B5] Dhillon G. S. Ahluwalia N. Honarbakhsh S. Graham A. Creta A. Abbass H. (2021). ‘Impact of adenosine on mechanisms sustaining persistent atrial fibrillation: Analysis of contact electrograms and noninvasive ECGI mapping data’. PLoS ONE 16 (3), 02489511–e249013. 10.1371/journal.pone.0248951 PMC7993562 33765054

[B6] Dhillon G. S. Schilling R. J. Honarbakhsh S. Graham A. Abbass H. Waddingham P. (2022). ‘Impact of pulmonary vein isolation on mechanisms sustaining persistent atrial fibrillation: Predicting the acute response’. J. Cardiovasc. Electrophysiol., 31, 903–912. 10.1111/jce.14392 32048786

[B7] Gao X. Lam A. G. Bilchick K. C. Darby A. Mehta N. Mason P. K. (2019). ‘The use of non-invasive mapping in persistent AF to predict acute procedural outcome’. J. Electrocardiol. 57, S21–S26. 10.1016/j.jelectrocard.2019.08.012 31474375

[B8] Guillem M. S. Climent A. M. Rodrigo M. Fernandez-Aviles F. Atienza F. Berenfeld O. (2016). ‘Presence and stability of rotors in atrial fibrillation: Evidence and therapeutic implications’. Cardiovasc. Res. 109 (4), 480–492. 10.1093/cvr/cvw011 26786157 PMC4777913

[B9] Haissaguerre M. Hocini M. Denis A. Shah A. J. Komatsu Y. Yamashita S. (2014) ‘Driver domains in persistent atrial fibrillation’, Circulation 130(7):530–538. 10.1161/CIRCULATIONAHA.113.005421 25028391

[B10] Haïssaguerre M. Hocini M. Sanders P. Takahashi Y. Rotter M. Sacher F. (2006). ‘Localized sources maintaining atrial fibrillation organized by prior ablation’. Circulation 113 (5), 616–625. 10.1161/CIRCULATIONAHA.105.546648 16461833

[B11] Haissaguerre M. Hocini M. Shah A. J. Derval N. Sacher F. Jais P. (2013). ‘Noninvasive panoramic mapping of human atrial fibrillation mechanisms: A feasibility report’. J. Cardiovasc. Electrophysiol. 24 (6), 711–717. 10.1111/jce.12075 23373588

[B12] Kis Z. Muka T. Franco O. H. Bramer W. M. De Vries L. J. Kardos A. (2017). ‘The short and long-term efficacy of pulmonary vein isolation as a sole treatment strategy for paroxysmal atrial fibrillation: A systematic review and meta-analysis’. Curr. Cardiol. Rev. 13 (3), 199–208. 10.2174/1573403x13666170117125124 28124593 PMC5633714

[B13] Li X. Almeida T. P. Dastagir N. Guillem M. S. Salinet J. Chu G. S. (2020). ‘Standardizing single-frame phase singularity identification algorithms and parameters in phase mapping during human atrial fibrillation’. Front. Physiology 11 (), 869. 10.3389/fphys.2020.00869 PMC7386053 32792983

[B14] Meng T. W. Choi G. P. T. Lui L. M. (2016). ‘TEMPO: Feature-endowed teichmüller extremal mappings of point clouds’. SIAM J. Imaging Sci. 9 (4), 1922–1962. 10.1137/15M1049117

[B15] Molero R. Soler Torro J. M. Martinez Alzamora N. M Climent A. Guillem M. S. (2021). ‘Higher reproducibility of phase derived metrics from electrocardiographic imaging during atrial fibrillation in patients remaining in sinus rhythm after pulmonary vein isolation’. Comput. Biol. Med. 139 (), 104934–104937. 10.1016/j.compbiomed.2021.104934 34688171

[B16] Narayan S. M. Krummen D. E. Shivkumar K. Clopton P. Rappel W. J. Miller J. M. (2012). ‘Treatment of atrial fibrillation by the ablation of localized sources: CONFIRM (conventional ablation for atrial fibrillation with or without focal impulse and rotor modulation) trial’. J. Am. Coll. Cardiol. 60 (7), 628–636. 10.1016/j.jacc.2012.05.022 22818076 PMC3416917

[B17] Podziemski P. Zeemering S. Kuklik P. van Hunnik A. Maesen B. Maessen J. (2018). ‘Rotors detected by phase analysis of filtered, epicardial atrial fibrillation electrograms colocalize with regions of conduction block’. Circulation. Arrhythmia Electrophysiol. 11 (10), e005858. 10.1161/CIRCEP.117.005858 PMC6553551 30354409

[B18] Rodrigo M. Climent A. M. Hernández-Romero I. Liberos A. Baykaner T. Rogers A. J. (2020). ‘Noninvasive assessment of complexity of atrial fibrillation: Correlation with contact mapping and impact of ablation’. Circ. Arrhythm. Electrophysiol. 13, 236–246. (March). 10.1161/CIRCEP.119.007700 PMC7508259 32078374

[B19] Rodrigo M. Climent A. M. Liberos A. Fernandez-Aviles F. Berenfeld O. Atienza F. (2017a). ‘Highest dominant frequency and rotor positions are robust markers of driver location during noninvasive mapping of atrial fibrillation: A computational study’. Heart 14 (8), 1224–1233. 10.1016/j.hrthm.2017.04.017 PMC5568422 28408329

[B20] Rodrigo M. Climent A. M. Liberos A. Fernandez-Aviles F. Berenfeld O. Atienza F. (2017b). ‘Technical considerations on phase mapping for identification of atrial reentrant activity in direct-And inverse-computed electrograms’. Circulation Arrhythmia Electrophysiol. 10 (9), e005008. 10.1161/CIRCEP.117.005008 28887361

[B21] Rogers J. M. Huang J. Smith W. M. Ideker R. E. (1999). ‘Incidence, evolution, and spatial distribution of functional reentry during ventricular fibrillation in pigs’. Circulation Res. 84 (8), 945–954. 10.1161/01.RES.84.8.945 10222342

[B22] Roney C. H. Pashaei A. Meo M. Dubois R. Boyle P. M. Trayanova N. A. (2019). ‘Universal atrial coordinates applied to visualisation, registration and construction of patient specific meshes’. Med. Image Anal. 55, 65–75. 10.1016/j.media.2019.04.004 31026761 PMC6543067

[B23] Vandersickel N. Van Nieuwenhuyse E. Van Cleemput N. Goedgebeur J. El Haddad M. De Neve J. (2019). ‘Directed networks as a novel way to describe and analyze cardiac excitation: Directed graph mapping’. Front. Physiology 10 (), 1138–1214. 10.3389/fphys.2019.01138 PMC6746922 31551814

