# Supplementary material for: AF driver detection in pulmonary vein area by electropcardiographic imaging: Relation with a favorable outcome of pulmonary vein isolation
Source: Front Physiol. 2023 Jan 30;14:1057700. doi: 10.3389/fphys.2023.1057700 (PMC9922892; doi:10.3389/fphys.2023.1057700)
Supplement: Supplementary file 2 [file Table1.docx]

Supplementary Material

# AF Simulation

Two Atrial fibrillation (AF) episodes 3 seconds long were simulated using a 3-dimensional realistic geometry of the human atria (284,578 nodes, 1,353,783 tetrahedrons and 673.4±130.3 µm between nodes)(Dössel *et al.*, 2012)(Rodrigo *et al.*, 2018)(Molero *et al.*, 2022). Action potentials at each node were simulated using a monodomain Koivumäki model (Koivumäki et. al., 2011). The resultant differential equations system was solved using Euler forward integration method on a graphic processor unit (NVIDIA GeForce RTX 2070) with a time step of 2ms. Gradients in different channel properties and inter-node conductivity were applied to modify the conduction velocity of the tissue. Reentries were induced in slow conduction areas by means of an S1-S2 stimulation protocol near the desired locations. (Rodrigo *et al.*, 2018). Full details about modeled currents, tissue conductivities, boundary conditions, and stimulation protocol are included in the simulation code as part of the supplementary materials. The epicardial electrical activity was calculated at a 1 mm distance from the external atrial surface by adding the dipole contributions of the transmembrane potentials. This approach works under the assumption of a homogeneous, unbounded, and quasistatic conducting medium between the surfaces (Romero *et al.*, 2019), and was implemented by applying equation 1 (Pedrón-Torrecilla *et al.*, 2016):

$$EGM=\sum_{r} \left( \frac{r}{r^{3}} \right)\cdot\nabla V_{m}$$

Where $r$ is the distance vector between nodes, $\nabla$ is the gradient, and $V_{m}$ is the transmembrane potential. The forward and inverse problems of electrocardiography were solved to obtain artificial ECG and ECGi signals out of the computed EGMs. A geometrical model of a human torso obtained from photogrammetry was used in this process. Before solving the inverse problem, 20 dBs of gaussian noise were added the ECG signals and a 0.5 to 20Hz bandpass filter was applied to remove this noise. Tikhonov 0-order regularization and L-curve optimization were applied to compute the inverse solution and the optimal λ value. In order to obtain smooth inverse computed Electrograms (iEGMs) and make them more similar to the ECGI signals from patients, we applied a bandpass filter around the dominant frequency at each atrial node [DFi -1, DFi+1]Hz. Finally, the signals where transform to 2D images following the procedure presented in the next section, and the Hilbert transform was applied to obtain phases.

# Rotor Detection Algorithm

The workflow of the proposed rotor detection algorithm is depicted in Figure 1. The input data for the this algorithm is a set of instantaneous 2D phase maps stacked in a volume. In this structure, the first 2 dimensions contain the spatial distribution of phases and the third dimension corresponds to time. PSs are initially detected by calculating the topological charge C at every pixel of the volume (see equation 1) (Guillem *et al.*, 2016)(Cluitmans *et al.*, 2018)(Podziemski *et al.*, 2018).

$$C= \oint\nablaϴ\cdot dr (1)$$

Where ϴ are the phase values in a closed curve r around each PS. If the absolute value of the topological charge C obtained was larger than a certain threshold, the pixel was considered as a possible phase singularity. Only the PSs presenting a gradual spatial progression between 0 and 2π in their surroundings were considered in the following steps (Rodrigo *et al.*, 2017). These PS were then stored as a volume of binary images encoding their spatiotemporal locations, where 1 indicated the presence of a PS and 0 the absence. Then, 3D binary dilation was performed using equation 2:

$$A\oplus B=\bigcup_{b\epsilon B} A_{b} (2)$$
Where $B$ is the 3D volume encoding the location of the PSs, $A$ is a 3 by 3 structural element containing ones and $A_{b}$ is a translated instance of A by $b$ pixels$.$ This operation allows connecting neighbouring PS and fill the gaps produced by misdetections. The dilation was always performed in the direction of

**Supplementary Figure 1.** 3D operations applied in the rotor detection algorithm and correspondent cross-sectional views: 1) The topological charge is calculated out of the input phase maps using 3D convolution operations. 2) A threshold is applied to obtain a binary 3D volume containing the space-temporal locations of phase singularities. 3) 3D dilation is applied on this volume in order connect close phase singularities and conform the space-temporal trajectories of rotors. 4) Skeleton of dilated volume is calculated and branch points are detected in order to remove crossing points in the trajectories. 5) Region growing is applied to the skeletonized volume in order to cluster the trajectories.

the filament in order to reduce interferences with other trajectories. However, occasionally dilation may also connect non-related filaments. The locations in the volume where two or more different trajectories are connected are called crossing points. In order to identify these crossings, binary skeletonization was applied by using the built-in MATLAB function bwmorph. Converging trajectories were split by deleting the crossing points. Then, region growing algorithm was applied to classify the resulting trajectories into different rotors.

Finally, the number of turns of each rotor was estimated. For that, the phase evolution throughout the spatiotemporal trajectory of the rotor was unwrapped in 4 different positions around each phase singularity. A first order polynomial curve was fitted to these phase evolutions and the mean squared error between the phase progression and the fitted model was computed in the four locations. The number of turns of the rotor was calculated as the range of phase covered in the position with the lowest mean squared error divided by 2π.

# Processing of geometrical models

Atrial geometries present complex anatomical structures. In order to avoid inhomogeneities in the distribution of nodes that may affect the accuracy of the inverse problem solution and posterior PS detection, we applied the framework presented in (Romero *et al.*, 2019). Additionally, this framework provides a way to reliably transform the electrophysiological data from 3D to 2D, allowing us to efficiently process the data with our rotor detection algorithm.

The first step consists in applying to the atrial geometry a remeshing algorithm presented in (Choi, Lam and Lui, 2015). This method parametrizes the geometrical model in a spherical domain (Praun and Hoppe, 2003). The data is then resampled in this domain in such a way that can be easily reorganized and uniformly distributed in a 2D grid. Specifically, the new set of nodes is obtained from a uniform triangulation of spherical mesh using an octahedron-based subdivision (Gu, Gortler and Hoppe, 2002). Then, the nodes obtained from the spherical parametrization of the atria, and the nodes obtained from the spherical parametrization of a 2D uniform grid are connected. The connectivity between the nodes is optimized using the convex hull algorithm in the spherical domain (Barber, Dobkin and Huhdanpaa, 1996). Finally, in order to obtain the electrophysiological data in the 2D grid domain, the data is interpolated in the uniform sampling domain (Amidror, 2002). This methodology is generally presented in figure 2, where the data can be directly open into a 2D grid.


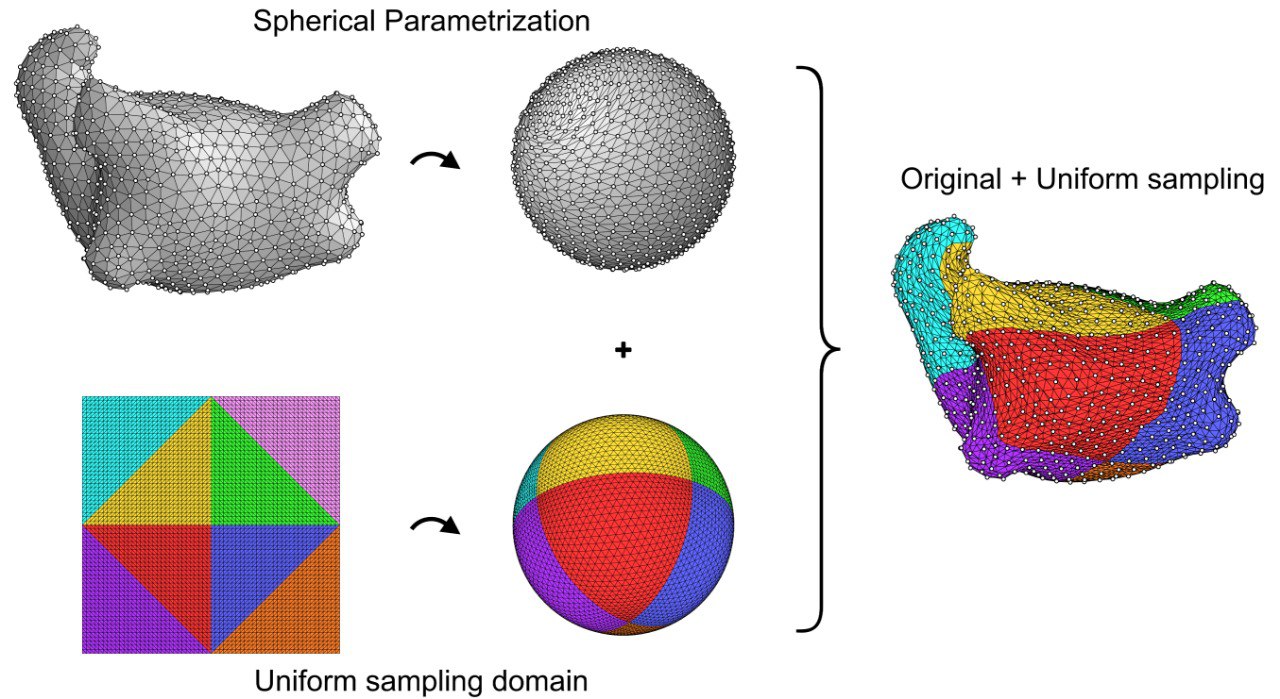


Supplementary Figure 2. Geometrical conformalization process (Romero *et al.*, 2019).

# Extended Results with Adenosine

In this section the results obtained in segments recorded during adenosine injection are presented and compared to the results obtained without adenosine. Specifically, the proportion of PS in the same 9 atrial regions were calculated. In figure 3 below, this proportions are presented using adenosine (first row of panels, 3.A and 3.B) and without adenosine (second row of panels, 3.C and 3.D). Additionally, the same analysis was performed considering all the PS (panels 3.A and 3.C) and removing all the PS belonging to rotors that spin for less than 1 turn ( panels 3.B and 3.D).

Similar regions with statistically significant differences in the proportion of PS and tendences are observed with and without adenosine. In total, only 3 different regions present statistically significant differences considering all the studied scenarios: PPVV, LAAP and SEPTUM. More importantly, the PPVV is the only region in which a significantly higher prevalence of reentries was observed in all the studied condition including adenosine injection. In fact, the difference between the studied groups is highlighted by its adenosine ( 12.09% vs 6.49%, p<0.01). Something noticeable when analyzing adenosine recordings, is that the application of a 1-turn filtering on the PSs seems to have a larger effect than in non adenosine recordings. For example, it can be observed how the differences in the proportion of PS in the PPVV increase more when applying the turn filtering. Additionally, the number of regions with significant differences is reduced from 3 to 1 in adenosine recordings.


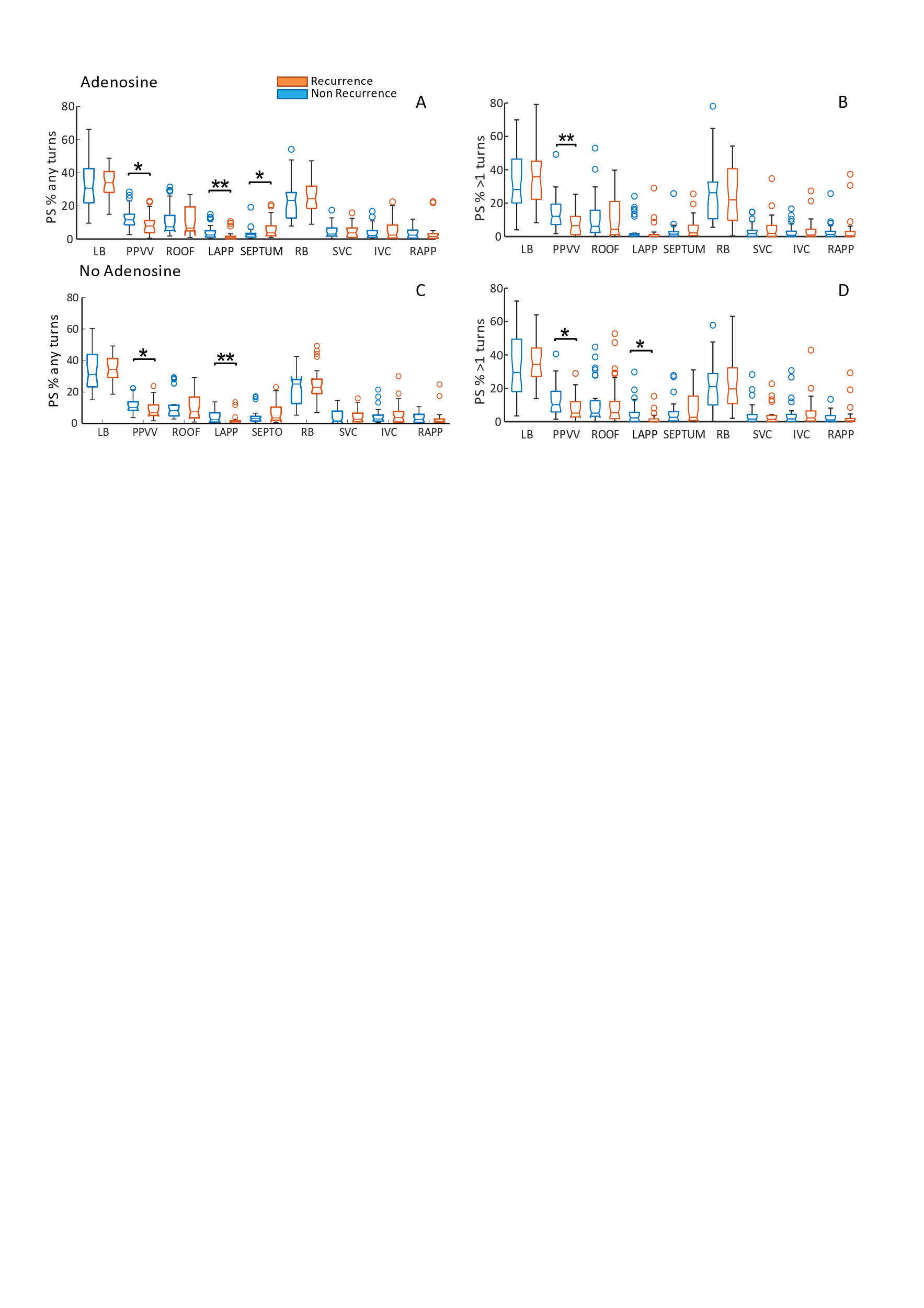
With the data collected it is very difficult to distinguish whether the mentioned differences observed when including adenosine are due the effect of adenosine accentuating the activity in the pro-arrhythmic areas, or it is simply because the absence of QRS complexes provide more reliable information of the atrial activity. However, the results obtained show that is still possible to see similar distribution of phase singularities along the atria and a constant higher prevalence of reentries in the PPVV in non recurrence patients without the use of any drug.

Supplementary Figure 3. Proportion of PSs per second detected in different regions in each studied group: in blue is presented the data from patients that remained in sinus rhythm 6 months after ablation (Non recurrence), and in orange patients that returned to any sort of arrhythmia (Recurrence). Panels A an d B present PS distributions in recordings obtained with adenosine injection and panels C and D without adenosine. On the other hand, A and C (left column) present the data when all the rotors, independently from their number of turns were considered in the analysis , and panels B and D (right column) when applying a threshold of 1 turn to consider the rotors*.*

# References

Amidror, I. (2002) ‘Scattered data interpolation methods for electronic imaging systems: a survey’, *Journal of Electronic Imaging*, 11(2), p. 157. doi: 10.1117/1.1455013.

Barber, C. B., Dobkin, D. P. and Huhdanpaa, H. (1996) ‘The Quickhull Algorithm for Convex Hulls’, *ACM Trans. Math. Softw.*, 22(4), pp. 469–483. doi: 10.1145/235815.235821.

Choi, P. T., Lam, K. C. and Lui, L. M. (2015) ‘FLASH: Fast landmark aligned spherical harmonic parameterization for genus-0 closed brain surfaces’, *SIAM Journal on Imaging Sciences*, 8(1), pp. 67–94. doi: 10.1137/130950008.

Cluitmans, M. *et al.* (2018) ‘Validation and opportunities of electrocardiographic imaging: From technical achievements to clinical applications’, *Frontiers in Physiology*, 9(SEP), pp. 1–19. doi: 10.3389/fphys.2018.01305.

Dössel, O. *et al.* (2012) ‘Computational modeling of the human atrial anatomy and electrophysiology’, *Medical and Biological Engineering and Computing*, 50(8), pp. 773–799. doi: 10.1007/s11517-012-0924-6.

Gu, X., Gortler, S. J. and Hoppe, H. (2002) ‘Geometry Images’, in *Proceedings of the 29th Annual Conference on Computer Graphics and Interactive Techniques*. New York, NY, USA: Association for Computing Machinery (SIGGRAPH ’02), pp. 355–361. doi: 10.1145/566570.566589.

Guillem, M. S. *et al.* (2016) ‘Presence and stability of rotors in atrial fibrillation: Evidence and therapeutic implications’, *Cardiovascular Research*, 109(4), pp. 480–492. doi: 10.1093/cvr/cvw011.

Koivumäki, J. T., Korhonen, T. and Tavi, P. (2011) ‘Impact of Sarcoplasmic Reticulum Calcium Release on Calcium Dynamics and Action Potential Morphology in Human Atrial Myocytes : A Computational Study’, 7(1). doi: 10.1371/journal.pcbi.1001067.

Molero, R. *et al.* (2022) ‘Effects of torso mesh density and electrode distribution on the accuracy of electrocardiographic imaging during atrial fibrillation’, *Frontiers in Physiology*, 13(August), pp. 1–10. doi: 10.3389/fphys.2022.908364.

Pedrón-Torrecilla, J. *et al.* (2016) ‘Noninvasive estimation of epicardial dominant high-frequency regions during atrial fibrillation’, *Journal of Cardiovascular Electrophysiology*, 27(4), pp. 435–442. doi: 10.1111/jce.12931.

Podziemski, P. *et al.* (2018) ‘Rotors Detected by Phase Analysis of Filtered, Epicardial Atrial Fibrillation Electrograms Colocalize With Regions of Conduction Block’, *Circulation. Arrhythmia and electrophysiology*, 11(10), p. e005858. doi: 10.1161/CIRCEP.117.005858.

Praun, E. and Hoppe, H. (2003) ‘Spherical Parametrization and Remeshing’, *ACM Trans. Graph.*, 22(3), pp. 340–349. doi: 10.1145/882262.882274.

Rodrigo, M. *et al.* (2017) ‘Technical considerations on phase mapping for identification of atrial reentrant activity in direct-And inverse-computed electrograms’, *Circulation: Arrhythmia and Electrophysiology*, 10(9). doi: 10.1161/CIRCEP.117.005008.

Rodrigo, M. *et al.* (2018) ‘Solving Inaccuracies in Anatomical Models for Electrocardiographic Inverse Problem Resolution by Maximizing Reconstruction Quality’, *IEEE Transactions on Medical Imaging*, 37(3), pp. 733–740. doi: 10.1109/TMI.2017.2707413.

Romero, I. H. *et al.* (2019) ‘Estimation of cardiac electrical activity by invasive and non-invasive mapping techniques Autor’.
