# Supplementary material for: AF driver detection in pulmonary vein area by electropcardiographic imaging: Relation with a favorable outcome of pulmonary vein isolation
Source: Front Physiol. 2023 Jan 30;14:1057700. doi: 10.3389/fphys.2023.1057700 (PMC9922892; doi:10.3389/fphys.2023.1057700)
Supplement: Supplementary file 4 [file DataSheet2.ZIP › SimulationCode/CodeSimulations.rtf]

Simulation Files

There are two simulations included: CarlosF_22_03_15_AblationOriginal_2StimAtriaSplit and CarlosF_22_03_09_AblationSmallRoofConstant. The first one corresponds to the AF episode where there are multiple rotors spread in the atria. The second one is driven by a single rotor close to the PPVV. In both, an ablation procedure is simulated. Here the information contained in the files used by the simulation cod is explained:
·	Aur_cond: Contains the conductivities between each node and 15 close neighbors.
·	Aur_init: File containing initial conditions of the simulation.
·	Aur_S1: This file stores the times of the stimulus in the fibrillation simulation. Each node has an excitation time. If a node is not going to be stimulated, it is labeled with -1.
·	Aur_vec: This file stores the neighbor nodes in the model. There is one line per node.
·	Aur_vels1: This file controls the conduction velocity in the model before the virtual ablation is applied. There is one number per node. These numbers multiply the basal conduction velocities.
·	Aur_vels2: Same information as Aur_vels1 but after the ablation. The velocities of the nodes around the PPVV were set to small values to simulate an ablation.
